# Association between the peripartum maternal and fetal telomere lengths and mitochondrial DNA copy numbers and preeclampsia: a prospective case–control study

**DOI:** 10.1186/s12884-022-04801-0

**Published:** 2022-06-13

**Authors:** Ruyi Zhang, Jiangbo Du, Zhendong Xiao, Yuan Jiang, Liang Jin, Qiao Weng

**Affiliations:** 1 Department of Obstetrics & Gynecology, Nanjing Drum Tower Hospital, the Affiliated Hospital of Nanjing University Medical School, Nanjing, 210008 China; 2 Department of Obstetrics & Gynecology, Drum Tower Clinical Medical College, Nanjing Medical University, Nanjing, 210008 China; 3 Department of Obstetrics & Gynecology, Nanjing Drum Tower Hospital Clinical College of Traditional Chinese and Western Medicine, Nanjing University of Chinese Medicine, Nanjing, 210008 China; 4 Department of Obstetrics & Gynecology, Nanjing Drum Tower Hospital Clinical College of Xuzhou Medical University, Nanjing, 210008 China; 5 Department of Epidemiology and Biostatistics, School of Public Health, Nanjing Medical University, Nanjing, 211166 China; 6 StateKey Laboratory of Reproductive Medicine, Nanjing Medical University, Nanjing, 211166 China

**Keywords:** Telomere length, Mitochondrial DNA copy number, Preeclampsia, Combined effect

## Abstract

**Purpose:**

To explore changes in telomere length (TL) and mitochondrial copy number (mtDNA-CN) in preeclampsia (PE) and to evaluate the combined effect of maternal TL and mtDNA-CN on PE risk.

**Methods:**

A case–control study of 471 subjects (130 PE cases and 341 age frequency matched controls with gestational age rank from 24 to 42 weeks) was conducted in Nanjing Drum Tower Hospital, Jiangsu Province of China. Relative telomere length (RTL) and mtDNA-CN were measured using quantitative polymerase chain reaction (qPCR), and PE risk was compared between groups by logistic regression analyses.

**Results:**

PE patients displayed longer RTL (0.48 versus 0.30) and higher mtDNA-CN (3.02 versus 2.00) in maternal blood as well as longer RTL (0.61 versus 0.35) but lower mtDNA-CN (1.69 versus 5.49) in cord blood (all *p* < 0.001). Exercise during pregnancy exerted an obvious effect of maternal telomere length prolongation. Multiparous women with folic acid intake during early pregnancy and those who delivered vaginally showed longer telomere length, while those factors imposed no or opposite effect on RTL in PE cases. Furthermore, RTL and mtDNA-CN were positively correlated in controls (in maternal blood *r* = 0.18, *p* < 0.01; in cord blood *r* = 0.19, *p* < 0.001), but this correlation was disrupted in PE patients in both maternal blood and cord blood. Longer maternal RTL and higher mtDNA-CN were associated with a higher risk of PE, and the ROC curve of RTL and mtDNA-CN for predicting PE risk presented an AUC of 0.755 (95% CI: 0.698–0.812).

**Conclusions:**

The interaction of TL and mtDNA-CN may play an important role in the pathogenesis of PE and could be a potential biomarker of PE risk.

**Supplementary information:**

The online version contains supplementary material available at 10.1186/s12884-022-04801-0.

## Introduction

Preeclampsia (PE), a serious multisystem disorder, is defined as a new onset of hypertension along with either proteinuria or end-organ dysfunction after 20 weeks of gestation [1]. With an incidence of 2–8% worldwide, PE is one of the leading causes of maternal and perinatal morbidity and mortality [1]. Although the pathophysiology of PE is not completely understood, early poor perfusion or placental ischaemia–reperfusion injury leading to increased oxidative stress has been widely accepted as one of the main pathological processes responsible for the development of PE [2, 3]. Some markers of oxidative stress are elevated in circulation in PE patients [4], but the specific changes in oxidative stress biomarkers in the context of PE are not well established, and insights into the relationship between oxidative stress markers and PE will help to illustrate the mechanisms of oxidative stress in the context of PE and to identify markers for disease prediction.

Telomere length (TL) and mtDNA copy number (mtDNA-CN) are emerging biomarkers of oxidative stress and have been related to various age-related diseases. They are highly susceptible to oxidative stress [5] and inflammation and have been suggested as sensitive indices of cellular oxidative stress, mitochondrial dysfunction, the ageing process, and age-related diseases [6].Recent research has indicated that telomeres and mitochondria are functionally linked and that telomere dysfunction could induce p53 to repress PGCs and result in metabolic and mitochondrial compromise, suggesting that disorder of the telomere-mitochondria axis may be an important and early event in biological ageing-related diseases [7, 8]. Lee et al. found that loss of the association between telomere length and mitochondrial DNA copy number may induce the initiation of colorectal carcinogenesis and that coregulation of telomeres and mitochondria may play an important role in the genesis and development of oxidative stress-related diseases [9]. However, evidence referring to TL and mtDNA-CN in the context of preeclampsia is scarce, and the results are mixed [10, 11]. Several studies have documented shorter TL in placentas from PE patients [12, 13], while another study did not find significant differences in placental TL between controls and PE cases and suggested a possible nuclear protective system, which might control TL via telomerase activity in PE placentas [14, 15]. A previous study was conducted with 50 patients with PE and 50 controls in Washington State. Harville et al. analysed telomere length in peripheral blood and found that women in the highest tertile had higher PE risk (OR 1.08) than those in the lowest tertile after adjusting for age, although the difference was not statistically significant [10], which may be explained by the limited sample size.

Therefore, we hypothesized that alterations in leukocyte TL and mtDNA-CN may reflect the cumulative exposure to oxidative stress and underlie the pathogenesis of PE. However, no previous study has been conducted on the association between TL and mtDNA-CN in PE patients. Here, we designed a case–control study with 130 PE cases and 341 controls in a Chinese population to investigate TL and mtDNA-CN alterations in maternal peripheral blood and cord blood in women with PE and to further explore their combined effect on PE risk. The results of the present study may aid in improving the current understanding of PE by identifying the joint involvement of TL and mtDNA-CN in PE pathogenesis.

## Materials and methods

### Study recruitment

PE cases and controls were recruited from Nanjing Drum Tower Hospital in Nanjing, Jiangsu Province of eastern China, between January 2019 and June 2020. According to the report of the American College of Obstetricians and Gynecologists’ Task Force on Hypertension in Pregnancy, PE is diagnosed by specialist doctors at admission [16]. During the same period, age-matched women who were healthy without any complications were recruited as controls. The inclusion criteria of the study were single viable pregnancy, with no restrictions on maternal age, BMI, or parity status. The exclusion criteria were multiple pregnancies; pregnancy with foetal anomalies; pregnancy with preexisting chronic diseases, such as chronic hypertension and diabetes mellitus; and other pregnancy complications, such as gestational diabetes, prelabour rupture of membranes, chorioamnionitis, and placental abruption. Finally, 130 cases and 341 controls were included with gestational age rank from 24 to 42 weeks. All subjects were informed about the study, and after signing the informed consent form, they were interviewed by well-trained interviewers with structured questionnaires. Data available in the questionnaire included data on demographics and lifestyle-related factors during pregnancy, such as education, folic acid intake, threatened abortion, physical activity, second-hand smoking, BMI at labour admission, and medical and reproductive characteristics. Delivery-related information such as gestational age, delivery mode, birth weight, and occurrence of postpartum haemorrhage (PPH) was obtained from obstetric records. Gestational age was calculated based on the last menstrual period or ultrasound-based estimated date of conception. Both physical activity and second-hand smoking were divided into four groups by levels.

Maternal peripheral venous blood samples were collected from 471 participants immediately before or during delivery. Paired umbilical cord blood samples were collected immediately after birth from the cord vein of newborns. Blood samples were shipped by cold chain equipment to the laboratory and stored until analysis. All procedures performed in studies involving human participants were in accordance with the ethical standards of the institutional and/or national research committee and the 1964 Declaration of Helsinki and its later amendments or comparable ethical standards. This study was approved by the ethics committees of Nanjing Drum Tower Hospital (No.2018–017).

### Measurement of relative telomere length and mitochondrial DNA copy number

Genomic DNA was extracted from leukocytes of maternal peripheral blood and cord blood. Telomere length and mtDNA-CN were analysed with modified quantitative polymerase chain reaction (qPCR) by a QuantStudio 7TM FlexReal-Time PCR System (Applied Biosystems). The ratio of telomere repeat copy number (T) to single-copy gene 36B4 number (S) was computed to reflect the relative telomere length (RTL). The primer sequences for telomere PCR were TEL1, 5′-GGTTTTTGAGGGTGAGGGTGAGGGTGAGGGTGAGGGT-3′, and TEL2, 5′-TCCCGACTATCCCTATCCCTATCCCTATCCCTATCCCTA-3′, and the single-copy gene (36B4) primer sequences were 36B4u, 5′-CAGCAAGTGGGAAGGTGTAATCC-3′, and 36B4d, 5′-CCCATTCTATCATCAACGGGTACAA-3′ [17]. We also determined mtDNA-CN as the ratio of mitochondrial-encoded NADH dehydrogenase-1(ND-1) to nuclear genes (haemoglobin subunit β, HGB) by simultaneous amplifications of ND1 and HGB genes. The primer sequences were as follows: ND1 forward 5'-CCCTAAAACCCGCCACATCT-3'; ND1 reverse 5'-GAGCGATGGTGAGAGCTAAGGT-3'; HGB forward 5'-GAAGAGCCAAGGACAGGTAC-3'; and HGB reverse 5'-CAACTTCATCCACGTTCACC-3' [18]. Reference DNA (pooled from 5 healthy controls) was used to generate a standard curve for quantification. After exclusion of outliers, average cycle threshold (Ct) values of the remaining samples were calculated. Each reaction system contained 10 μl SYBR ® Green PCR Master Mix (Applied Biosystems) with a final DNA concentration of 5 ng/μl. All samples were assayed in duplicate, and three quality controls were employed in each plate to analyse variability. qPCRs were executed by investigators blinded to clinical data and disease status. RTL and mtDNA-CN were calculated based on Cawthon’s formula [17, 18]:

$$2^{-\;(\Delta Ct1-\Delta Ct2)}=\:2.^{-\Delta\Delta Ct}$$

### Statistical analysis

All statistical analyses were performed with Stata version 15.1 (Stata Corp, College Station, TX). Sample characteristics are described as the mean (SD), median (IQR) or percentage. Pearson χ^2^ test was used to test differences in categorical variables (maternal age group, education, folic acid intake, threatened abortion, physical activity, second-hand smoke exposure status, BMI at delivery and mode of delivery) between cases and controls. T tests and Wilcoxon rank tests were employed to compare the differences in normally distributed and nonparametric continuous variables, respectively, between cases and controls. The Wilcoxon signed rank test was utilized for the comparisons of RTL and mtDNA-CN between matched maternal blood and cord blood. The Kruskal–Wallis rank test was used to compare nonparametric variables among groups. Correlations of RTL and mtDNA-CN in PE cases and controls were analysed by Spearman’s rank correlation. To estimate the relative association between preeclampsia and levels of maternal RTL and mtDNA-CN, we categorized maternal RTL and mtDNA-CN into two groups according to their median distribution. The odds ratios (ORs) and 95% confidence intervals (95% CIs) of RTL and mtDNA-CN associated with PE risk were calculated by logistic regression analyses. Then backwards stepwise logistic regression was carried out to explore the independent factors associated with PE risk. The receiver operating characteristic (ROC) curve and the area under the ROC curve (AUC) were calculated to assess use of maternal RTL and mtDNA-CN as possible markers o PE risk. A two-sided *P* < 0.05 was considered statistically significant, and *p* values with significance are marked in bold in tables.

## Results

### Baseline characteristics of PE cases and controls

**Fig. 1:**
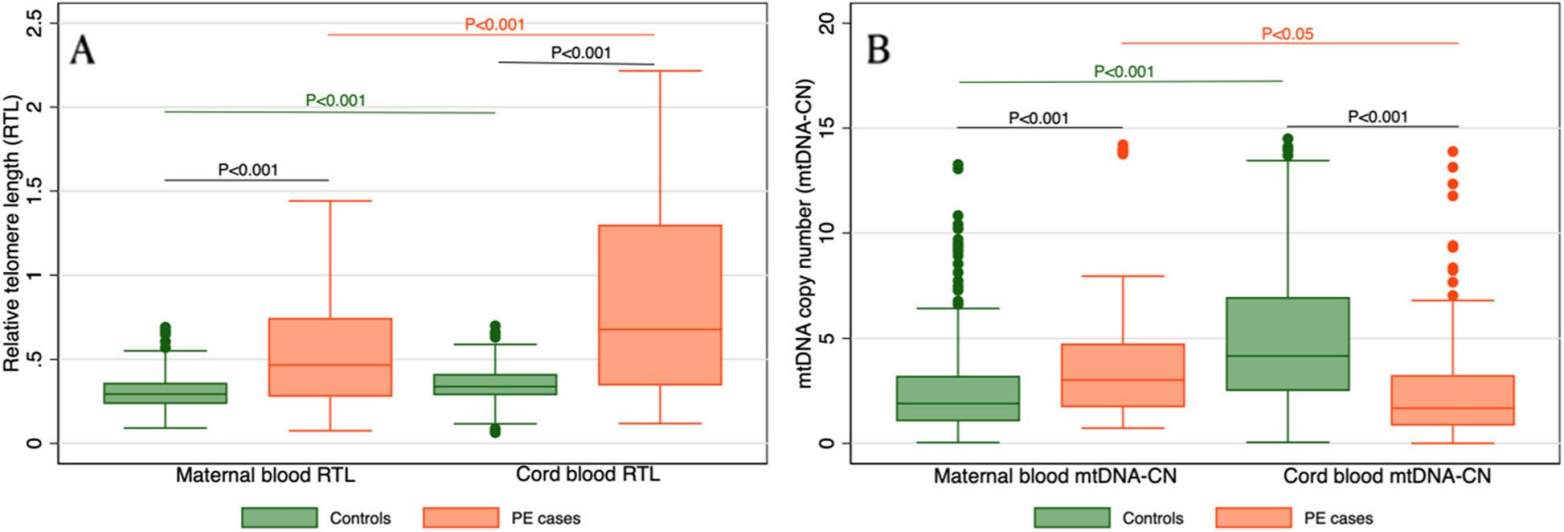
Relative telomere length (RTL) and mitochondrial DNA copy number (mtDNA-CN) in maternal blood and cord blood. **A** RTL from controls and PE cases in maternal blood and cord blood. **B** mtDNA-CN from controls and PE cases in maternal blood and cord blood

Women with PE tended to be less educated, had more gravidities, had a lower folic acid intake rate, had a higher threatened abortion rate in early pregnancy, had more second-hand smoke exposure, had a younger gestational age, had a higher BMI at delivery, had a higher caesarean section rate, had a lower birth weight, and had a higher PPH incidence but a higher physical activity level than controls (*p* < 0.05). No significant differences were found for parity between cases and controls. Maternal RTL was significantly longer in PE patients than in controls (median: 0.48 versus 0.30; *p* < 0.001), and this trend was also observed in cord blood (median: 0.61 versus 0.35; *P* < 0.001). Maternal mtDNA-CN of PE cases was significantly higher than that of controls (median: 3.02 versus 2.00; *p* < 0.001); however, in cord blood, lower mtDNA-CN was found in PE cases (median: 1.69 versus 5.49; *p* < 0.001) (Table 1, Fig. 1).

**Table 1:** Characteristics of study participants in PE cases and controls

	**PE cases**	**Controls**	***p* value**
**N**	130	341	
**Maternal age (years)**			
< 25	14(10.77)	27(7.92)	0.052
25 ~ 29	56(43.08)	179(52.49)	
30 ~ 34	38(29.23)	104(30.50)	
> = 35	22(16.92)	31(9.09)	
**Education**			
Low	46(35.38)	59(17.30)	** < 0.001**
Middle	53(40.77)	140(41.06)	
High	31(23.85)	142(41.64)	
**Gravidity**	1.98 + -0.09	1.68 + -0.05	**0.002**
**Parity**	1.22 + -0.04	1.14 + -0.02	0.05
**Folic acid intake**			
None	33(25.38)	29(8.50)	** < 0.001**
Sometimes	29(22.31)	87(25.51)	
Nearly everyday	68(52.31)	225(65.98)	
**Threatened abortion**			
No	102(78.46)	313(91.79)	** < 0.001**
Yes	28(21.54)	28(8.21)	
**Exercise**			
None	42(32.31)	184(53.96)	** < 0.001**
1–2 times per week	34(26.15)	98(28.74)	
3–4 times per week	32(24.62)	38(11.14)	
5–7 times per week	22(16.92)	21(6.16)	
**Second hand smoking**			
None	64(49.23)	235(68.91)	** < 0.001**
Less than 0.5 h a day	27(20.77)	79(23.17)	
0.5–1 h a day	27(20.77)	15(4.40)	
More than 1 h a day	12(9.23)	12(3.52)	
**Gestational age**	37.20 + -0.28	39.19 + -0.13	** < 0.001**
**BMI at delivery**			
Quantile 1	30(23.08)	89(26.25)	** < 0.001**
Quantile 2	20(15.38)	96(28.32)	
Quantile 3	30(23.08)	87(25.66)	
Quantile 4	50(38.46)	67(19.76)	
**Delivery mode**			
Vaginal delivery	45(34.62)	289(84.75)	** < 0.001**
Caesarean section	85(65.38)	52(15.25)	
**Birth weight**	2827.08 + -69.85	3303.19 + -30.26	** < 0.001**
**Postpartum Hemorrhage**			
No	78(60.00)	279(81.82)	** < 0.001**
Yes	52(40.00)	62(18.18)	
**Maternal blood RTL**	0.48(0.27–0.77)	0.30(0.24–0.36)	** < 0.001**
**Cord blood RTL**	0.61(0.35–1.28)	0.35(0.29–0.42)	** < 0.001**
**Maternal blood mtDNA-CN**	3.02(1.83–4.89)	2.00(1.15–3.31)	** < 0.001**
**Cord blood mtDNA-CN**	1.69(0.91–3.60)	5.49(2.89–10.15)	** < 0.001**

### Comparisons of maternal RTL and mtDNA-CN in subgroups of PE cases and controls

As shown in Table 2, median RTL and mtDNA-CN in maternal blood were not influenced by maternal age, education, previous pregnancy, threatened abortion, preterm delivery, BMI at delivery, birthweight, or occurrence of PPH. Participants who engaged in more exercise during pregnancy tended to have longer maternal RTL (median: 0.76 versus 0.35 in cases and 0.32 versus 0.30 in controls, both *p* < 0.05). In controls, women who were multiparous, women with folic acid intake during early pregnancy and those who delivered vaginally showed longer telomere length, while those factors imposed no or the opposite effect on RTL in PE cases. Smoke exposure did not impact RTL in cases or controls but displayed an effect of increasing mtDNA-CN in controls. Less exercise was associated with higher mtDNA-CN in controls; while in PE patients, it did not show any impact on mtDNA-CN.

**Table 2:** Comparisons of maternal RTL and mtDNA-CN in subgroups of PE cases and controls

	**PE cases**					**Controls**				
**Characteristics**	**n**	**RTL**	***p* value**	**mtDNA-CN**	***p* value**	**n**	**RTL**	***p* value**	**mtDNA-CN**	***p* value**
**Overall**	**130**					**341**				
**Maternal age (year)**			0.1096		0.3575			0.8179		0.475
< 25	14	0.56(0.42–0.77)		1.70(1.29–3.66)		27	0.30(0.25–0.36)		2.35(1.58–3.93)	
25 ~ 29	56	0.44(0.26–0.73)		3.17(1.52–5.02)		179	0.29(0.23–0.37)		1.86(1.02–3.09)	
30 ~ 34	38	0.41(0.26–0.82)		3.30(2.06–4.63)		104	0.31(0.25–0.35)		2.05(1.19–3.21)	
> = 35	22	0.69(0.46–0.83)		2.94(2.09–5.49)		31	0.32(0.23–0.40)		2.23(1.03–4.42)	
**Education**			0.1522		0.0972			0.928		0.9138
Low	46	0.62(0.29–0.85)		2.26(1.38–4.77)		59	0.30(0.23–0.40)		2.17(1.05–3.52)	
Middle	53	0.45(0.25–0.74)		3.42(2.12–5.32)		140	0.30(0.24–0.37)		2.01(1.16–3.54)	
High	31	0.41(0.28–0.62)		3.03(1.92–4.16)		142	0.30(0.25–0.36)		1.98(1.15–3.25)	
**Previous pregnancy**			0.1946		0.3067			0.0572		0.2386
No	51	0.43(0.27–0.70)		2.96(1.66–4.30)		189	0.29(0.24–0.36)		2.04(1.27–3.31)	
Yes	79	0.55(0.28–0.82)		3.38(1.84–5.28)		152	0.31(0.24–0.38)		1.96(1.03–3.37)	
**Parity**			0.1385		0.5436			**0.0029**		0.555
Primiparous	102	0.45(0.27–0.73)		3.02(1.72–4.73)		291	0.29(0.24–0.36)		2.00(1.16–3.45)	
Multiparous	28	0.59(0.31–0.87)		3.21(1.88–5.54)		50	0.34(0.30–0.39)		2.01(1.04–3.05)	
**Folic acid intake**			**0.0281**		0.6406			**0.0369**		0.5704
No	33	0.67(0.39–0.88)		3.51(1.72–4.51)		29	0.26(0.22–0.32)		2.38(1.10–4.62)	
Yes	97	0.45(0.27–0.73)		2.94(1.83–5.02)		312	0.30(0.25–0.37)		1.99(1.15–3.25)	
**Threatened abortion**			0.9862		0.1643			0.3249		0.6863
No	102	0.48(0.27–0.82)		2.90(1.84–4.51)		313	0.30(0.24–0.36)		1.99(1.15–3.52)	
Yes	28	0.47(0.29–0.73)		4.01(1.64–6.11)		28	0.30(0.26–0.37)		2.12(1.12–2.96)	
**Exercise**			** < 0.001**		0.5665			**0.0309**		** < 0.001**
None or seldom	76	0.35(0.24–0.49)		3.01(1.92–5.04)		282	0.30(0.24–0.36)		2.18(1.31–3.57)	
Often	54	0.76(0.51–0.97)		3.04(1.57–4.67)		59	0.32(0.23–0.48)		1.03(0.95–2.12)	
**Second hand smoking**			0.0761		0.8427			0.8967		**0.0393**
None	64	0.45(0.28–0.76)		2.84(1.61–4.72)		235	0.30(0.24–0.36)		1.96(1.05–3.37)	
Less than 0.5 h a day	27	0.37(0.22–0.70)		3.17(1.85–6.22)		79	0.29(0.24–0.36)		1.84(1.01–2.86)	
0.5–1 h a day	27	0.60(0.34–0.86)		2.57(1.96–4.87)		15	0.33(0.22–0.43)		2.67(2.16–5.57)	
More than 1 h a day	12	0.71(0.33–0.81)		3.64(1.29–5.77)		12	0.28(0.22–0.38)		2.38(1.84–3.41)	
**Preterm delivery**			0.9717		0.3489			0.5142		0.3924
No	86	0.47(0.28–0.73)		2.75(1.76–4.74)		313	0.30(0.24–0.37)		1.98(1.12–3.22)	
Yes	44	0.49(0.27–0.81)		3.67(1.92–5.04)		28	0.29(0.24–0.36)		2.67(1.29–3.69)	
**BMI at delivery**			0.2521		0.4288			0.2482		0.8637
Quantile 1	30	0.70(0.26–0.91)		3.78(1.86–4.88)		89	0.29(0.23–0.34)		1.99(1.28–3.00)	
Quantile 2	20	0.52(0.28–0.77)		2.01(1.38–3.20)		96	0.30(0.24–0.37)		2.20(1.18–3.37)	
Quantile 3	30	0.46(0.37–0.79)		3.45(2.21–4.87)		87	0.30(0.24–0.37)		1.77(1.05–3.32)	
Quantile 4	50	0.43(0.26–0.66)		3.01(1.92–5.04)		67	0.31(0.25–0.37)		1.87(1.03–4.21)	
**Delivery mode**			**0.0105**		0.2416			**0.0308**		0.1702
Vaginal delivery	45	0.40(0.26–0.62)		3.02(1.66–4.16)		289	0.30(0.24–0.37)		1.96(1.05–3.37)	
Caesarean section	85	0.57(0.29–0.89)		3.01(1.88–5.31)		52	0.28(0.23–0.33)		2.25(1.42–3.25)	
**Low birth weight**			0.311		0.7459			0.5765		0.1952
No	93	0.45(0.27–0.73)		2.95(1.84–5.00)		318	0.30(0.24–0.37)		1.98(1.10–3.30)	
Yes	37	0.65(0.29–0.82)		3.51(1.68–4.89)		23	0.29(0.23–0.36)		2.69(1.38–3.52)	
**Postpartum hemorrhage**			0.3484		0.459			0.4309		0.4442
No	78	0.45(0.27–0.73)		3.17(1.89–4.88)		279	0.30(0.24–0.37)		2.03(1.15–3.58)	
Yes	52	0.55(0.28–0.81)		2.62(1.51–5.00)		62	0.29(0.24–0.35)		1.93(1.07–2.86)	

### Association between RTL and mtDNA-CN in maternal blood and cord blood

**Fig. 2:**
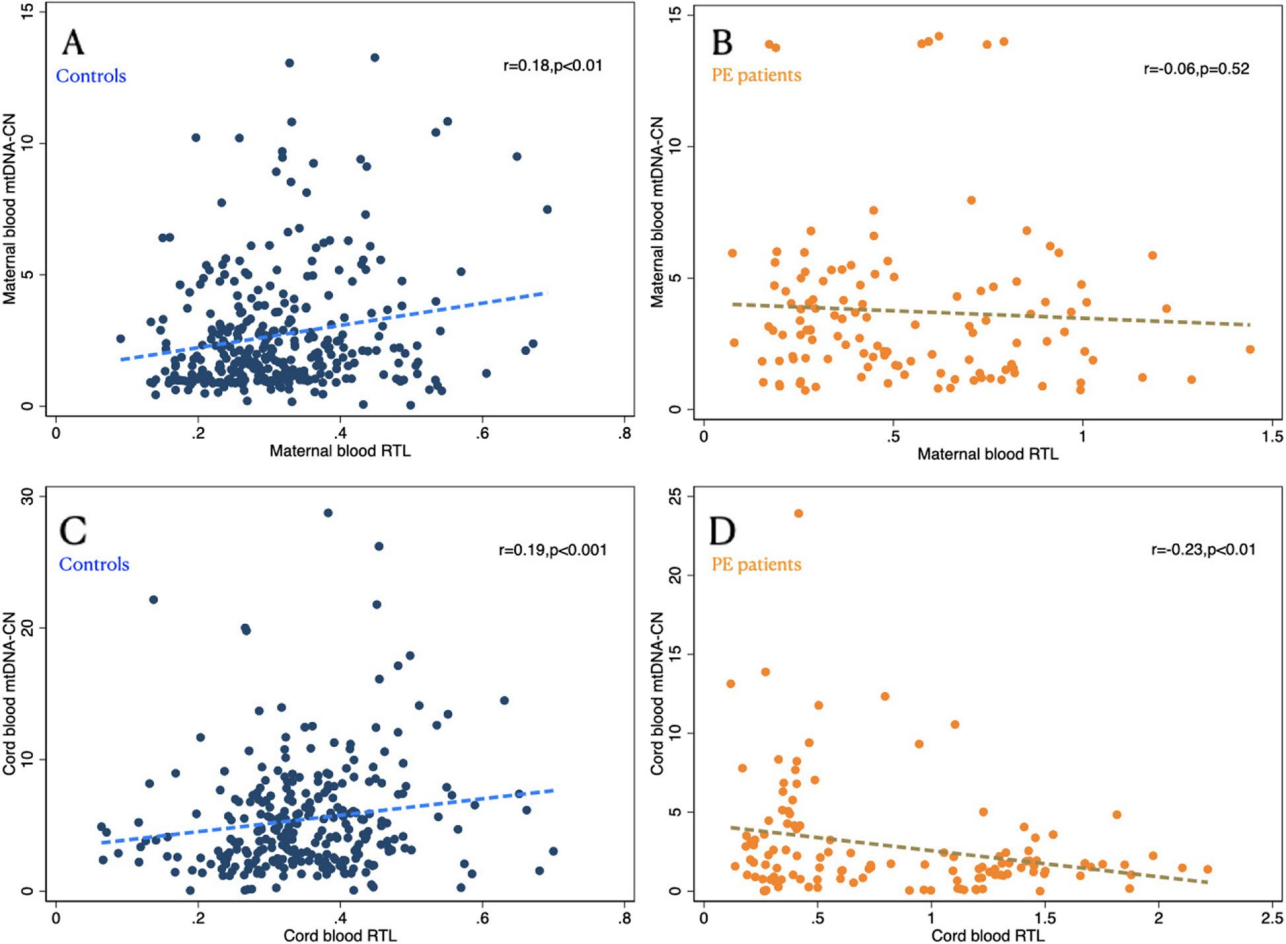
Association of RTL and mtDNA-CN in maternal blood and cord blood from controls and PE cases. **A** RTL and mtDNA-CN in maternal blood from normal pregnancy. **B** RTL and mtDNA-CN in maternal blood from PE patients. **C** RTL and mtDNA-CN in cord blood from normal pregnancy. **D** RTL and mtDNA-CN in cord blood from PE patients

As shown in Fig. 2, a positive correlation between maternal RTL and mtDNA-CN was determined in women with a normal pregnancy (*r* = 0.18, *p* < 0.01, Fig. 2A), but this correlation disappeared in PE patients (*p* = 0.52, Fig. 2B). We also found a positive correlation between RTL and mtDNA-CN in cord blood among controls (*r* = 0.19, *p* < 0.001, Fig. 2C), while a negative correlation was observed in PE cases (*r* = -0.23, *p* < 0.01, Fig. 2D).

### PE risk according to different categories of maternal RTL and mtDNA-CN

Logistic regressions were used to estimate factors associated with PE risk. In the multivariate logistic model, maternal RTL and mtDNA-CN were strong predictors of PE risk (Table 3). To stratify the analysis of the association of PE risk with maternal RTL and mtDNA-CN, we separately categorized maternal RTL and mtDNA-CN into two groups according to their median values. When compared to those in the lower(shorter) group, a significantly elevated risk of PE was observed in both higher(longer) groups (RTL: aOR:2.02, 95% CI: 1.22–3.32, *p* = 0.006; mtDNA-CN: aOR: 2.14, 95% CI: 1.26–3.63, *p* = 0.005) after adjustment for maternal age, education, gravidity, parity, folic acid intake, threatened abortion, second-hand smoking, physical exercise and BMI at delivery (Table 4).

**Table 3:** Multivariate logistic model for identifying factors associated with PE risk

PE risk	OR	95%CI	p
**Maternal RTL**	118.13	22.20–628.65	** < 0.001**
**Maternal mtDNA-CN**	1.1	1.01–1.20	**0.032**
**Maternal age (Ref: < 25)**			
25 ~ 29	0.99	0.39–2.57	0.992
30 ~ 34	0.89	0.33–2.43	0.824
> = 35	0.82	0.26–2.65	0.744
**Education(Ref: low)**			
Middle	0.6	0.31–1.14	0.12
High	0.5	0.25–1.01	0.054
**Gravidity**	1.22	0.90–1.67	0.204
**Parity**	1.03	0.46–2.33	0.935
**Folic acid intake(Ref: none)**			
Sometimes	0.57	0.24–1.35	0.204
Nearly everyday	0.59	0.27–1.28	0.183
**Threatened abortion(Ref: no)**	3.06	1.53–6.13	**0.002**
**Exercise(Ref: none)**			
1–2 times per week	1.47	0.79–2.75	0.222
3–4 times per week	1.66	0.74–3.72	0.216
5–7 times per week	1.44	0.52–4.01	0.481
**Second hand smoking(Ref: none)**			
Less than 0.5 h a day	1.38	0.74–2.56	0.314
0.5–1 h a day	3.05	1.24–7.51	**0.016**
More than 1 h a day	1.82	0.58–5.67	0.302
**BMI at delivery**	1.05	0.99–1.12	0.114

**Table 4:** Odds ratios (OR) and 95% Confidence Interval (CI) for preeclampsia according to categories of maternal RTL and mtDNA-CN

Variables	PE cases	Controls	OR(95%CI) ^a^	*p* ^a^
	***N* (%)**	**N (%)**		
Maternal RTL				**0.006**
Low(≤ 0.314)	40(31.25)	195(57.18)	1	
High(> 0.314)	88(68.75)	146(42.82)	2.02(1.22–3.32)	
Maternal mtDNA-CN				**0.005**
Low(≤ 2.20)	46(36.51)	186(55.03)	1	
High(> 2.20)	80(63.49)	152(44.97)	2.14(1.26–3.63)	

^a^Logistic regression analysis, adjusted for age, BMI, gravidity, parity, folic acid intake, threatened abortion, second hand smoke, physical activity and education

### Predictive abilities of maternal RTL and mtDNA-CN for PE

**Fig. 3:**
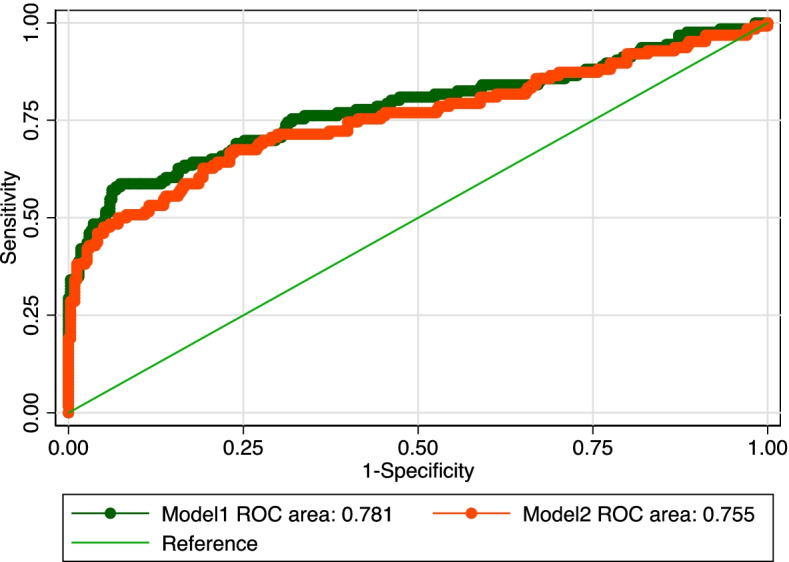
ROC curves for PE risk prediction. Model 1 incorporated all related maternal characteristics from Table 1; model 2 included only maternal RTL and mtDNA-CN

We applied logistic regression models with backwards stepwise procedures to investigate the independent factors associated with PE risk (Fig. 3). Model 1 incorporated all the maternal characteristics associated with PE from Table 1, while Model 2 included only maternal RTL and mtDNA-CN. The ROC curves and AUCs of the two models were calculated. The results revealed that maternal RTL and mtDNA-CN were strong predictors of PE risk. Maternal RTL, mtDNA-CN, threatened abortion and second-hand smoking during pregnancy jointly showed significant predictive value for PE risk, with an AUC of 0.781 (95% CI: 0.726–0.836) in Model 1, while Model 2 with RTL and mtDNA-CN only presented an AUC of 0.755 (95% CI: 0.698–0.812). There was no significant difference between these two models (*p* > 0.05).

## Discussion

Given the dearth of findings on the role of RTL and mtDNA-CN in the pathophysiology of PE, this study aimed to compare RTL and mtDNA-CN in PE patients and normal pregnant controls and to evaluate the predictive value of maternal RTL and mtDNA-CN for PE risk. Our study demonstrated that PE patients displayed longer RTL and higher mtDNA-CN in maternal blood as well as longer cord blood RTL but lower cord blood mtDNA-CN than normal pregnant women. Multiparity, exercise during pregnancy and first trimester folic acid intake exerted lengthening effects on maternal telomere length. Furthermore, RTL and mtDNA-CN were positively correlated in healthy pregnant women and fetuses, while this correlation was disrupted in PE cases. We also found that longer maternal RTL and higher mtDNA-CN were associated with a higher risk of PE and that the combination of maternal RTL and mtDNA-CN was effective in predicting PE risk. To our knowledge, this was the first study to examine the predictive ability of the combination of maternal RTL and mtDNA-CN for PE risk.

Previous studies reported shorter telomeres in placental samples from pregnancies complicated with PE and suggested that abnormal telomere homeostasis was closely related to the pathogenesis of PE and suggested that TL could be a potential biomarker of PE [12]. Evidence has supported the consistency of telomere length from different tissues; thus, easily accessible leukocytes are widely employed as a substitute for tissue in telomere length measurement [19–22]. However, research on peripheral leukocyte TL and PE is scarce. As TL shortening has been associated with various ageing-related diseases, it is somewhat surprising that we found that longer leukocyte TL was associated with PE in the current study. More physical activity among our PE patients may partially explain this inconsistency; most of our PE patients were more likely to follow doctors’ advice during pregnancy and maintained good lifestyles, although they had been in an initially abnormal state. Evidence supports that the telomere length of leukocyte cells is positively associated with a healthy lifestyle, and physical activity may confer protection against telomere shortening [23–25]. Thus, the modified lifestyle may lengthen TL to some extent to compensate for TL attrition when most PE cases in our study were mild and well controlled, with imperceptible shortening; however, this effect still needs to be confirmed and clarified in our future studies. Furthermore, although telomeres shorten throughout human life in cells of most human tissues, telomere length is heavily confounded by, among other factors, the variable levels of telomerase activity—and hence variable capacities for telomere length replenishment—in stem cells. Stem cells can constantly renew somatic tissue cells. Telomerase is enriched in germline lineage cells and embryonic stem cells [26–29]; previous data have indicated that telomerase reverse transcriptase (TERT) and telomerase activities were significantly higher in preeclampsia placentae than in the control group placentae according to different gestational ages [30, 31], which may cause a lengthening effect of TL in PE cases as well. Additionally, despite the consistency of telomere length among tissues, leukocyte TL may not completely reflect TL changes in the placenta in PE cases. More studies are warranted to elucidate the correlations between leukocyte TL and placental TL throughout the progression of PE.

The contribution of mitochondrial dysfunction to the pathogenesis of preeclampsia has been proposed by Torbergsen et al. [32] and Widschwendter et al. [33], the latter indicating that defects in trophoblastic mitochondria may be the initial step in the pathophysiological process of PE. In this study, we found that higher mtDNA-CN was associated with higher PE risk, which corroborates previous findings [34]. Previous data also reported that the odds of preeclampsia were positively associated with maternal blood mtDNA-CN [35]. These results further support the idea that elevated peripheral blood mtDNA-CN may serve as a risk marker for PE. It has been demonstrated that mtDNA is highly susceptible to oxidative stress and mitochondrial damage, as reflected by changes in mtDNA-CN that may alter mitochondrial gene expression and cause oxidative phosphorylation deficiency and a surge of ATP by glycolysis [36]. In addition, oxidative stress, an important pathogenesis pathway involved in preeclampsia [37], may alter mitochondrial function and increase mtDNA-CN through several mechanisms, and it is possible that elevated systematic levels of reactive oxygen species (ROS) may impair or disrupt cellular components such as mitochondrial lipid membranes [38]. ROS may also influence mitochondrial function by impairing DNA and damaging the electron transport chain, and a compensatory response to this cellular stress may lead to an increase in mtDNA-CN [36, 39]. This result is highlighted by experimental animal studies demonstrating increased mitochondrial damage and mtDNA-CN with increasing exposure to pro-oxidants [38]. These data together suggest that the association of increased mtDNA-CN with preeclampsia is biologically plausible. However, several other studies reported inconsistent findings. Some have shown an increase in mtDNA-CN in PE maternal blood and placentas [34, 40], while others have shown no changes in placentas of PE patients [41] or reduction of mtDNA-CN in PE peripheral blood during the first trimester of pregnancy [42]. The heterogeneity in results between studies may be related to population diversity, lifestyle modifications, exposure levels, time windows and mitochondrial DNA compensation [43, 44].

The initial setting of TL represents a critically important characteristic of an individual’s telomere biology system [45]. In our study, we found that the RTL of cord blood was longer than that of maternal blood in both controls and PE cases. However, we did not observe a reverse association between age and RTL in pregnant women. Our narrow age range may have accounted for this lack of association; the rate of changes in telomere length varies with age and is greatest in childhood and elderly individuals but relatively stable in adults [46, 47]. In addition, only approximately 10 percent of women were 35 years or older and less than 10 percent were younger than 25 years in our study, so the small sample in those subgroups may yield some deviation. The above reasons may dilute the adverse effects of ageing on telomere length in the current study. Recent studies emphasized that telomere length was associated with diet and lifestyle determinants [48–50]. In our study, we also found that both folic acid intake and regular exercise prolonged maternal TL in normal pregnancy, which further supports the idea that TL could be adjusted by modifying lifestyles. This result underlined that TL was sensitive enough to reflect alterations in oxidative stress in pregnant women and could be a reliable biomarker of oxidative stress during pregnancy.

Although mtDNA-CN has been directly related to obesity in the paediatric population [51], we did not observe this effect in our study. Higher mtDNA-CN was shown to be associated with less exercise and more second-hand smoke exposure in normal pregnancy in this study, while in PE patients, this impact was disrupted. The excessive oxidative stress generated by less exercise and more second-hand smoke exposure may lead to increased mtDNA-CN synthesis as a compensatory mechanism to ensure cell survival. In addition to population diversity, variations in levels and duration of exposure to oxidative stress [43, 52] caused by genetic factors as well as environmental factors have a dual influence on mtDNA-CN. Mild oxidative stress may first respond to increased energy demands by increasing mtDNA-CN [39, 53], while continued high exposure to oxidative stress may induce mtDNA damage and result in decreased or limited replication of mtDNA due to increased abundance of defective mitochondria [39, 44]. This duality in response to mild vs. excessive oxidative stress might also explain previous mixed findings on mtDNA content. It is worth noting that umbilical cord blood mtDNA-CN in PE cases was much lower than that of controls, which was in contrast to our result of maternal blood; in all probability, pregnancy is a period when fetuses are extremely susceptible to oxidative stress as at this time, mitosis is highly active, with a result that mitochondria in cord blood of PE patients exceeded the extent of compensation and showed a decrease in copy number. This finding is in line with a recent study that observed that air pollution exposure during pregnancy was associated with decreased mtDNA content in cord blood and suggested heightened sensitivity to oxidative stress during the specific prenatal window of life stage [54].

In our control subjects, there were positive correlations between mtDNA-CN and telomere length in maternal blood and cord blood, suggesting coregulation of telomeres and mitochondrial function in mothers and fetuses in normal pregnancy, even though the correlations were weak. Telomere length and mtDNA-CN have largely been examined as independent contributors to oxidative stress-related diseases, yet there is growing evidence that these two markers are functionally linked or that at least combining them may better predict disease development. Several recent studies concerning school-age children and healthy adults as well as elderly women reported a positive association between mtDNA-CN and telomere length [55–58]. Studies involving animal models and cell culture experiments have also shown that telomere dysfunction is associated with abnormal mitochondrial biogenesis and function [59, 60]. However, limited evidence with a small sample size [61] has documented the dependence between these two biomarkers in pregnant women. Therefore, we found correlations between RTL and mtDNA-CN when they were measured both in maternal and cord blood during normal pregnancy. The mechanism of this association remains to be determined; mitochondrial effects of p53 activation from telomere dysfunction [7] and TERT effects on mtDNA repair may be involved [62]. Telomere shortening can reciprocally lead to cellular mitochondrial endangerment and diminished mitochondrial biogenesis via diminution of PGC-1α, the master regulator of mitochondrial biogenesis [7]. In addition, TERT, a catalytic subunit of telomerase with a canonical role in telomere maintenance, contains both nuclear localization signals and mitochondrial targeting sequences and might be transported from nuclei to mitochondria under increased oxidative stress conditions to regulate mitochondrial function and protect mtDNA from oxidative damage [63]. However, this association was not observed in PE cases, implying that the pathways shared by regulating telomere length and mitochondrial biogenesis might be disturbed by the pathophysiology of PE. The present findings provide evidence that telomeres and mitochondria are coregulated in normal pregnant women and their fetuses, and this coregulation is interrupted when PE occurs, with increased RTL and mtDNA-CN being associated with increased PE risk. In this sense, the pathophysiology of PE may play a role in the mechanisms regulating the association between telomere length and mtDNA-CN, and the complex interplay between TL and mtDNA-CN could be a potential effective predictive factor for PE risk. Considering this, we first investigated the cumulative effect of TL and mtDNA-CN on PE risk. As a result, we found that the combination of maternal leukocyte mtDNA-CN and RTL can effectively predict the risk of PE, contributing to recent investigations concerning improvements in PE prediction models. As the relations between RTL and mtDNA-CN in our study were not very strong and the results were from blood taken at the moment of delivery, it would be more useful to assess these variables in earlier pregnancy maternal blood samples to improve predictive value. Further studies are still needed to verify our results, and more precise knowledge of the regulatory pathways governing the interaction of RTL and mtDNA-CN with the PE process is also necessary to delineate both its onset and pathogenesis.

Our study has some strengths. We were the first to investigate the combined effect of maternal RTL and mtDNA-CN on PE prediction and the first to reveal the disruption of positive dependence between these two biomarkers participating in the process of PE even without knowing whether the relationship was causal or simply an association. Second, many lifestyle factors, such as smoking status, folic acid intake, exercise during pregnancy and BMI before delivery, which may influence RTL and mtDNA-CN, were assessed and adjusted for in the present study to examine the independent effect of RTL and mtDNA-CN on PE risk. Moreover, unlike other studies regarding BMI and pregnancy, we chose to use BMI before delivery rather than prepregnancy BMI because this variable would have more of an impact on RTL and mtDNA-CN measured in our study, as it takes into account weight gain during pregnancy.

Several limitations also need to be acknowledged. First, we only measured RTL and mtDNA-CN before delivery, and the alterations of RTL and mtDNA-CN in leukocytes during different stages of pregnancy and disease progression remain unclear. Second, our study was restricted to Han Chinese individuals; the generalizability of the findings to other ethnic cohorts needs further evaluation. Third, due to the sample size, our study treated all PE cases as one group; types of PE need to be investigated separately, as early-onset preeclampsia has different pathogeneses than late-onset PE. Fourth, although we incorporated a series of lifestyle-related factors influencing RTL and mtDNA-CN into our study, many other factors, such as stress, environmental pollutants and detailed dietary habits, were not included, as they are difficult to measure objectively. Finally, we only carried out association analyses among RTL, mtDNA-CN, and risk of PE. The underlying mechanisms that account for pathways of leukocyte mtDNA content and RTL effect PE pathophysiology need further investigation.

## Conclusions

In conclusion, the absence of a positive correlation between RTL and mtDNA-CN in our study may partially explain the initiation or progression of PE pathogenesis; additionally, maternal RTL and mtDNA-CN before delivery were positively associated with the risk of PE, suggesting that increased levels of maternal RTL and mtDNA-CN are risk factors for PE and that their combined effect may have a predictive efficacy for PE risk. This study demonstrates the contribution of interplay between RTL and mtDNA-CN to pathogenesis or development of PE and opens a new perspective for PE prediction.

## Supplementary information

**Additional file 1.** Data of study. All data that has been collected and analyzed present in this file.

## Data Availability

All data generated or analyzed during this study are included in this published article and its supplementary information files.

## Abbreviations

TL: Telomere Length
mtDNA-CN: Mitochondrial Copy Number
PE: Preeclampsia
RTL: Relative Telomere Length
qPCR: Quantitative Polymerase Chain Reaction
PPH: Postpartum Hemorrhage
ORs: Odds Ratios
ROC: Receiver Operating Characteristic
AUC: Area Under the ROC Curve
TERT: Telomerase Reverse Transcriptase
